# Monitoring and Detection of Insecticide Resistance in *Spodoptera frugiperda* (Lepidoptera: Noctuidae): Evidence for Field-Evolved Resistance in Egypt

**DOI:** 10.3390/insects15090705

**Published:** 2024-09-16

**Authors:** Moataz A. M. Moustafa, Nourhan A. El-Said, Nawal AbdulAziz Alfuhaid, Fatma M. A. Abo-Elinin, Radwa M. B. Mohamed, Ahmed A. A. Aioub

**Affiliations:** 1 Department of Economic Entomology and Pesticides, Faculty of Agriculture, Cairo University, Giza 12613, Egypt; moat_mon@agr.cu.edu.eg (M.A.M.M.); nourhanadel158@gmail.com (N.A.E.-S.); fatma.mohamed57689@gmail.com (F.M.A.A.-E.); radwamahamed599@gmail.com (R.M.B.M.); 2 Department of Biology, College of Science and Humanities, Prince Sattam Bin Abdulziz University, Al-Kharj 11942, Saudi Arabia; n.alfuhaid@psau.edu.sa; 3 Plant Protection Department, Faculty of Agriculture, Zagazig University, Zagazig 44511, Egypt

**Keywords:** *Spodoptera frugiperda*, insecticide resistance, resistance monitoring, synergists, detoxification enzymes

## Abstract

**Simple Summary:**

The fall armyworm, *Spodoptera frugiperda*, represents a major threat to cereal crops. Therefore, this study aimed to detect the resistance of the second-instar larvae of *S. frugiperda* in two Egyptian field strains to eight insecticides. The results showed that emamectin benzoate, had the highest toxicity against the susceptible and the two field populations of *S. frugiperda* larvae followed by spinosad > lufenuron > diflubenzuron > cypermethrin > profenfos > methoxyfenozide > Bacillus thuringiensis. Additionally, resistance to cypermethrin followed by spinosad and lufenuron was observed in Fayoum field population. Thus, the combined use of synergists with cypermethrin, spinosad, and lufenuron showed synergistic effects against *S. frugiperda* larvae. This study underscores the need for pest management strategies based on regional resistance profiles and biochemical characteristics.

**Abstract:**

*Spodoptera frugiperda* (J.E. Smith) (Noctuidae: Lepidoptera) is a notable insect pest that invades major cereal crops, causing significant damage and loss. Resistances of 2nd instar larvae of two Egyptian field populations of *S. frugiperda*, collected from the Fayoum and Giza governments, were measured against eight insecticides, including traditional insecticides (profenofos and cypermethrin), bio-insecticides (emamectin benzoate, spinosad, and *Bacillus thuringiensis*), and insect growth regulators (IGRs) (lufenuron, diflubenzuron, and methoxyfenozide). In addition, the synergistic effects of three synergists (Piperonyl butoxide (PBO), diethyl maleate (DEM), and triphenyl phosphate (TPP) were assessed, and the activities of detoxification enzymes (acetylcholine esterase (AChE), cytochrome P-450 (CYP-450), carboxylesterase (CarE), and glutathione-s-transferase (GST) were also determined. Resistance surveillance revealed that the Fayoum field population showed moderate resistance to cypermethrin (RR = 5.75-fold), followed by spinosad (RR = 2.62-fold), and lufenuron (2.01-fold). On the other hand, the Giza population exhibited significant resistance to cypermethrin only (RR = 3.65-fold). Our results revealed that emamectin benzoate was the most effective insecticide, with an LC_50_ value of 0.003 mg/L for the Fayoum population and 0.001 mg/L for the Giza population, compared to the susceptible strain (0.005 mg/L). Among the biological insecticides, *Bacillus thuringiensis* was the least toxic insecticide of all the tested strains. Synergism assays indicated that DEM and TPP had the most synergistic effect on spinosad (SR = 8.00-fold for both), followed by PBO (SR = 5.71-fold) for the Fayoum population, compared with spinosad alone. The assay of detoxification enzymes showed that GST activity significantly (*p* < 0.05) increased in the two field strains compared to the susceptible strain. However, no significant changes were observed among the tested strains in CYP-450, CarE, or AChE. The findings of this study provide substantial insights into tracking and managing the development of insecticide resistance in *S. frugiperda* in Egypt.

## 1. Introduction

The fall armyworm, *Spodoptera frugiperda* (J.E. Smith) (Lepidoptera: Noctuidae), is native to the Neotropical areas of Central and South America [1,2]. The first notable *S. frugiperda* infestations in Africa were found in 2016 in southeastern Nigeria [3]. Its propagation has expanded over sub-Saharan Africa [4], India [5], Asia [2,6], and Australia [7], and is currently thought to be the world’s most significant corn pest. In 2018, it was classified by the Food and Agriculture Organization in Egypt as a worldwide pest that needs to be quarantined. The first infestation was recorded in 2019 in Upper Egypt’s maize farms [8]. *S. frugiperda* is a polyphagous and well-known pest that feeds on over 350 plant species [9]. Its distribution is continually expanding, and with the ongoing impacts of climate change, its pest status is expected to become even more severe [10,11]. The economic relevance of *S. frugiperda* is attributed to its robust flight (up to 500 km before oviposition), extensive migration, great dispersal capacity, and high reproduction rate [12]. *S. frugiperda* was brought to the Eastern Hemisphere and swiftly expanded from Western Africa to Southeast Asia. All these characteristics have drawn attention from all around the world [2].

The critical phase of *S. frugiperda* larvae is the caterpillar, which feeds on tender leaf whorls, ears, and tassels, severely reducing grain output and causing considerable damage to maize crops [13,14]. Young maize seedlings can have their entire base cut through by late larval instars, leading to the destruction of the entire plant [15]. Mitchell [16] approximated the yield lost due to *S. frugiperda* in the USA to be around US$300 million per year, and this number is expected to reach US$500 million or more during significant epidemic years. Moreover, it is estimated that *S. frugiperda* costs sub-Saharan Africa up to US$13 billion a year, in damage to sugarcane, sorghum, rice, and corn [17]. Furthermore, in 2017, losses from maize were predicted to have cost Zambia and Ghana US$198 million and US$284 million, respectively. The total loss across 12 African nations was estimated to have cost US$2.5–6.3 billion [18]. In Egypt, *S. frugiperda* damages about 78.89% of maize harvests yearly [19]. Therefore, *S. frugiperda* is considered one of the world’s most devastating insects, with the potential to cause severe damage to economically important crops.

Due to its high adaptability, *S. frugiperda* is well known for developing resistance to insecticides [20,21]. The first reports of *S. frugiperda* population resistance were made in 1976 in Georgia [22] and in 1978 in Alabama, where typical chemical treatments and synthetic pyrethroids were unable to provide effective control [23]. Moreover, this pest exhibited resistance to six modes of action groups in the Americas and to at least 29 insecticidal active ingredients by 2017 [24,25,26]. Furthermore, resistance to zeta-cypermethrin, chlorantraniliprole, flubendiamide, methomyl, spinetoram, chlorpyrifos, deltamethrin, permethrin, triflumuron, and thiodicarb was discovered in a single population of *S. frugiperda* in Puerto Rico [27]. Additionally, El-Sayed, et al. [28] reported that the enhanced metabolic detoxification of insecticides is one major mechanism through which *S. frugiperda* develops resistance to these insecticides. In insects, the three stages of cellular detoxification are identified as phase I, phase II (metabolizing enzymes), and phase III (transporters) [29]. Phase I is carried out by CYP-450 monooxygenases and carboxylesterases (CarE), phase II by glutathione-s-transferases (GSTs), and phase III by ATP-binding cassette transporters (ABC) [30].

Several studies demonstrated that increased enzymatic activity is one of the basic mechanisms of insect resistance that could hydrolyze or block pesticides [31,32]. Synergists, such as synergists, in combination with chemical pesticides may help manage, and postpone the development of insect resistance by overcoming the metabolic resistance mechanism [33]. Piperonyl butoxide (PBO), diethyl maleate (DEM), and triphenyl phosphate (TPP) are frequently employed as inhibitors of monooxygenases, glutathione-s-transferases, and esterases, respectively [34]. These synergists are used to track resistance mechanisms and to enhance the effectiveness of insecticides [35].

Resistance monitoring at the biochemical level should be a part of any integrated pest management (IPM) program [36]. Therefore, it is imperative to examine the biochemical profile of the susceptible laboratory strain and field populations of *S. frugiperda* to establish a foundation for understanding the biochemical basis of resistance and to develop effective control strategies.

The current research aims to assess the toxicity of eight different insecticides belonging to three classes, viz., traditional insecticides (profenofos and cypermethrin), bio-insecticides (emamectin benzoate, spinosad, and *Bacillus thuringiensis (BT)*), and insect growth regulators (IGRs) (lufenuron, diflubenzuron, and methoxyfenozide) to the 2nd instar larvae of a susceptible strain and two Egyptian field populations of *S. frugiperda* collected from Fayoum and Giza governorates. In addition, the efficacy of PBO, DEM, and TPP, as synergists, in conjunction with cypermethrin, spinosad, and lufenuron was assessed in the Fayoum population. Furthermore, the activities of CYP-450s, CarE, GSTs, and acetylcholinesterase (AchE) enzymes in the susceptible strain and the two field populations were also measured and correlated with insecticide toxicity.

## 2. Materials and Methods

### 2.1. Insects

Larvae of the susceptible strain of *S. frugiperda* were reared in the laboratory on castor bean leaves as described by Moustafa et al. [37], Moustafa et al. [38] and Awad et al. [39] without exposure to pesticides for over ten generations. Moths were kept in a glass jar with a 10% sugar solution [40]. Two field populations of *S. frugiperda* were collected from maize cultivars in Fayoum (29°18′28″ N and 30°24″ E.) and Giza (30°0′47.0016″ N and 31°12′31.8708″ E.) during the 2023 season. Both populations were maintained under laboratory conditions of 25 ± 1 °C temperature, 60–70% relative humidity, and a 16:8 h (L:D) photoperiod for one generation. The F1 2nd instar larvae were then used for the toxicity bioassay experiments.

### 2.2. Insecticides and Chemicals

The tested insecticides are listed in Table 1. The substrates, reagents, and synergists, including piperonyl butoxide (PBO), triphenylphosphate (TPP), and diethyl maleate (DEM), were procured from Sigma Aldrich, Darmstadt, Germany.

### 2.3. Toxicity Bioassay

The toxicity of the insecticides against the second instar larvae of both susceptible and field strains was assessed under 25 ± 1 °C temperature and 60–70% relative humidity using the leaf dipping technique [30,41]. Five concentrations ranging from 100 to 2.5 mg/L for conventional insecticides, 8000 to 0.00025 mg/L for bio-insecticides, and 2 to 0.00125 mg/L for IGR were prepared. The castor bean leaves (0.5 cm × 0.5 cm) were immersed in each concentration for 20 s and then left to dry in the air [37,42]. Four replicates, each containing 25 larvae, were used for each concentration [43]. Water-dipped leaves were used in control groups. Treatments and control were repeated twice to guarantee the accuracy of the findings. Larvae that cannot move upon touch with a brush were defined as dead. The mortality was evaluated 96 h after treatment to determine the resistance ratio (RR) and calculate LC values.

### 2.4. Synergism Assay

Synergists (PBO as a CYP-450 inhibitor, DEM as a GST inhibitor, and TPP as an esterase(s) inhibitor) were used in conjunction with cypermethrin, spinosad, and lufenuron to determine the efficacy of these combinations on the Fayoum population. Synergists were mixed with insecticide concentrations at a rate of 100 mg/L. The synergistic ratios (SR) were calculated as indicated by Qie, Lu, Aioub, Li, Wu, and Hu [34].

### 2.5. Enzyme Assays

#### 2.5.1. Enzyme pPreparation

A sample of 50 mg of second instar larvae from both the susceptible and field strains wasere homogenized in 0.1 M phosphate buffer at a 1:10 (W) ratio [44]. Each population was replicated five times.

#### 2.5.2. Cytochrome P-450 (CYP-45) Assay

CYP-450 activity was determined as outlined by Hansen and Hodgson [45]. The larvae were homogenized in a phosphate buffer (pH 7.8) and then centrifuged at 15,000× *g* for 15 min at 4 °C. In each well of the microplate, a solution comprising 2 mM p-nitroanisole (100 μL) and enzyme stock solution (90 μL) was added and incubated for 2 min at 27 °C. To start the reaction, 10 μL of 9.6 mM NADPH was added. CYP-450 activity was monitored for 15 min at a wavelength of 405 nm.

#### 2.5.3. The Carboxylesterase (CarE) Assay

Carboxylesterase activity was assessed following the method outlined by Van Asperen [46]. The spectrophotometric measurement of α-naphthyl acetate hydrolysis was conducted at wavelengths of 600 nm. Total CarE activity was determined by utilizing standard curves of α-naphthol and protein content.

#### 2.5.4. Glutathione S- Transferase (GST) Assay

GST activity was assessed following the procedure described by Habig et al. [47], employing 1-chloro-2,4-dinitrobenzene (CDNB) as the substrate. The larvae were homogenized in phosphate buffer (pH 6.5) and then centrifuged at 12,000× *g* for 15 min at 4 °C. The reaction mixture consisted of 10 μL of supernatant, 25 μL of 30 mM CDNB, and 25 μL of 50 mM glutathione (GSH). GST activity was monitored at 340 nm in kinetic mode over a 5-min period.

#### 2.5.5. Acetylcholinesterase (AChE) Assay

AChE activity was estimated as outlined by Simpson et al. [48]. ATChI (Acetylthiocholine iodide) was used as the substrate. The reaction solution consisted of enzyme solution, phosphate buffer, substrate, and reagent (DTNB; dDithio-bis-nitro benzoic acid). The reduction in ATChI concentration was measured at 405 nm.

### 2.6. Statistical Analysis

Probit analysis was employed to estimate the lethal concentrations (LCs) and their 95% confidence limits (CLs) [49]. Enzymatic activity data were statistically analyzed using one-way ANOVA followed by Tukey’s Honestly Significant Difference (HSD) test for significance, performed with GraphPad Prism (v. 9.3).

## 3. Results

### 3.1. Susceptibility of Field Populations of Spodoptera frugiperda to Insecticides

The toxicity of the tested insecticides to the two field populations of *S. frugiperda* is summarized in Table 2 and Table 3. The recorded LC_50_ values were (0.005, 0.003, and 0.001 mg/L) for emamectin benzoate, (0.01, 0.028, and 0.01 mg/L) for spinosad, and (0.08, 0.16, and 0.05 mg/L) for lufenuron in the susceptible strain, Fayum, and Giza field populations, respectively. Moreover, no resistance (RR < 2-fold) was observed for profenofos, emamectin benzoate, diflubenzuron, methoxyfenozide, or *Bacillus thuringiensis* in the Fayoum or Giza populations, and to spinosad or lufenuron in the Giza population. On the other hand, a moderate level of resistance was detected for cypermethrin (RR = 5.75 and 3.65-fold) in the Fayoum and Giza populations, respectively, and for lufenuron (RR = 2.01) and spinosad (RR = 2.62) in the Fayoum population only.

### 3.2. Synergism Studies

The synergistic effects of PBO, DEM, and TPP on the toxicities of cypermethrin, spinosad, and lufenuron to the Fayoum population are given in Table 4, Table 5 and Table 6. PBO exhibited the most significant synergistic effect on cypermethrin in the susceptible strain and Fayoum population, with synergism ratios of 1.30 and 1.68-fold, respectively. In addition, resistance in the Fayoum population was suppressed when DEM and TPP were added to cypermethrin, with synergism ratios of 1.41 and 1.44, respectively. However, combinations of cypermethrin and DEM or TPP had no synergistic effect in the susceptible strain (Table 4).

As shown in Table 5, adding TPP to spinosad reduced its LC_50_ to the susceptible strain from 0.01 to 0.004 mg/L, with a synergistic ratio of 2.50-fold, compared to 0.66 and 1.00-fold with PBO and DEM, respectively. On the other hand, adding PBO, DEM, and TPP significantly enhanced the toxicity of spinosad to the Fayum field population, with synergism ratios of 5.71-, 8.00-, and 8.00-fold, respectively, compared with spinosad alone.

The data in Table 6 indicated that PBO caused slight synergism with lufenuron in the susceptible strain (SR = 1.23), whereas DEM and TPP had no synergistic effect (SR = 0.88 and 0.54, respectively). Regarding the Fayum field population, PBO and DEM also exhibited slight synergism (SR = 1.14-fold for both), while TPP showed no synergism with Lufenuron (SR = 0.96-fold).

### 3.3. Detoxification Enzymes Assay

**Figure 1:**
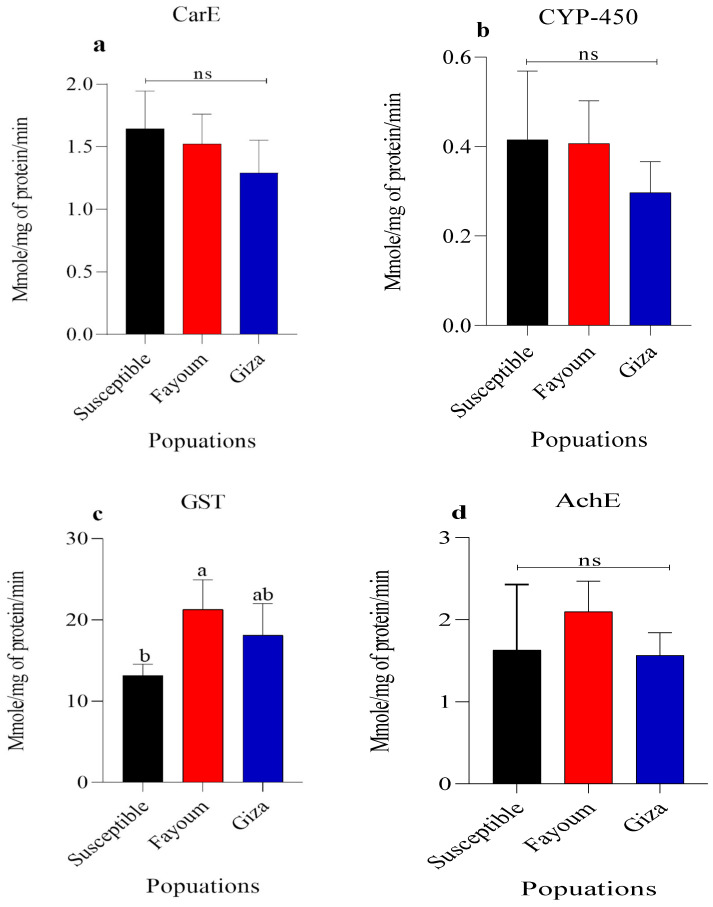
Carboxylesterases (CarE; **a**), cytochrome p-450 (CYP-450; **b**), glutathione-s-transferase (GST; **c**) and acetylcholinesterase (AChE; **d**) activities in the 2nd instar larvae of a susceptible strain and two field populations of *S. frugiperda*. Each column represents the mean ± SEM of three independent experiments. *p*-values were determined using Tukey’s HSD test. Different letters above the bar indicate significant differences (*p* < 0.05).

Enzymatic activities of AChE, CarE, CYP-450, and GST in the treated 2nd instar larvae of susceptible strains, Fayum and Giza field populations of *S. frugiperda* are shown in Figure 1. Our results indicated no significant differences in the activities of AChE, CarE, and CYP-450 across the tested populations, compared to the susceptible strain. However, a significant (*p* > 0.05) increase in GST activities was recorded in the Fayoum, and Giza populations (21.29 and 18.11 Mmole/mg of protein/min), compared to the susceptible strain (13.18 Mmole/mg of protein/min).

## 4. Discussion

The misuse of chemical insecticides worldwide leads to the development of resistance, exacerbating outbreaks caused by invasive pests such as *S. frugiperda*. Additionally, invasive pests may inherently possess resistance to specific insecticides [50]. *S. frugiperda* management involves the use of various groups of insecticides, including organophosphorus, pyrethroids, insect growth regulators (IGRs), and bioinsecticides. Consequently, resistance to insecticides has emerged in this pest species [29,50]. Consequently, it’s essential to investigate the historical trends of insect resistance to diverse insecticides, including *S. frugiperda*, to monitor shifts in tolerance and promptly identify any emerging issues. Furthermore, monitoring insecticide resistance yields valuable insights into the natural reactions of *S. frugiperda* populations to insecticides and helps pinpoint regions with consistent resistance patterns, aiding in the development of targeted management strategies [51]. Hence, monitoring insecticide usage is deemed essential in Integrated Pest Management (IPM) initiatives [52], and emerges as a significant component of resistance management [53,54].

Our study evaluated the susceptibility of the 2nd instar larvae of a susceptible strain and two field populations (Fayoum and Giza) of *S. frugiperda* to two insecticides (profenofos and cypermethrin), three bio-insecticides (emamectin benzoate, spinosad, and *Bacillus thuringiensis*), and three insect growth regulators (IGRs) (lufenuron, diflubenzuron, and methoxyfenozide) with different modes of action. Additionally, the effects of synergists (PBO, DEM, and TPP) on the toxicity of cypermethrin, spinosad, and lufenuron to the susceptible and Fayoum strains of *S. frugiperda* were investigated. Moreover, the activities of the relevant enzymes were examined to identify the mechanism of resistance.

Our data showed that emamectin benzoate, spinosad, and lufenuron had the highest toxicity against the susceptible and the two field populations of *S. frugiperda* larvae compared to the other tested insecticides. This finding aligns with the research conducted by El-Sayed, Ibrahim, Elsobki, and Aioub [28], which demonstrated that the LC_50_ value of emamectin benzoate to the 3rd instar larvae of *S. frugiperda* larvae was 0.029 mg/L. Moreover, spinosad and emamectin benzoate were more toxic than conventional insecticides to *S. frugiperda* larvae [55,56]. Additionally, the LC_50_ value of lufenuron against *S. frugiperda* larvae was 0.99 mg/L 24 h after treatment [57]. Based on the resistance ratio, emamectin benzoate, is the most effective insecticide against the two tested field populations, which is consistent with other reports [58,59]. Nevertheless, the two tested field populations exhibited resistance to cypermethrin compared to other insecticides. This is likely because bioinsecticides have not been widely used in Egypt, so their toxicity to *S. frugiperda* remains high with little resistance. Additionally, this resistance may be attributed to the use of less effective conventional insecticides, the natural selection process in the field, or the migration of the pest from regions with higher resistance levels [51].

Insecticide resistance involves two primary mechanisms: upregulation of detoxification enzyme activity and reduced sensitivity of the target site [60]. Synergists are substances that either temporarily or permanently interfere with enzymes, preventing them from functioning correctly, and thus act as insecticide synergists [61]. These synergists are crucial in addressing resistance issues related to insecticide application [62,63].

Our data demonstrate that the synergists PBO, DEM, and TPP substantially enhanced the effectiveness of cypermethrin, spinosad, and lufenuron (except for TPP with lufenuron) in the Fayoum population of *S. frugiperda*. This enhancement is likely due to the synergistic effect observed when these compounds are combined with insecticides. This effect is usually linked to the inhibition of various detoxifying enzymes essential for insects, as these enzymes play an essential role in their defense against insecticides [64]. The analogous result reported by Hafeez, et al. [65] revealed that the indoxacarb toxicity to *S. frugiperda* was enhanced by PBO. Interestingly, PBO, DEM, and TPP increased the cypermethrin and spinosad toxicity against fourth larval instars of *Spodoptera littoralis* when compared to the efficacy of the insecticides tested individually [28]. Notably, PBO exhibited greater synergistic effects with spinosad compared to TPP and DEM in *Spodoptera exigua* [66]. In addition, the synergistic effect of PBO and DEM significantly increased the toxicity of cypermethrin in both *Apis mellifera* and *Musca domestica* [67]. Furthermore, PBO, DEM, and TPP demonstrated synergistic effects, increasing the effectiveness of cypermethrin by 1.5-, 1.7-, and 2.3-fold, respectively, against *Tuta absoluta* (Meyrick) [68]. Wang et al. [69] demonstrated that in the Taian population, PBO enhanced the toxicity of spinosad more than in the Xinjiang population, with synergistic ratios of 2.0 and 4.7, respectively, 48 h after treatment against *Helicoverpa armigera*. Moreover, the toxicity of spinosad, when combined with PBO and DEM, showed an increase in both susceptible and resistant strains of *Musca domestica* compared to spinosad alone [70].

Elevated activity levels of detoxification enzymes in certain populations are a key factor contributing to insecticide resistance [71,72]. The current study found insignificant differences in AChE, CarE, and CYP-450 activities between the susceptible strain and the field populations of *S. frugiperda*. This finding aligns with the research conducted by Zhang, et al. [73], which indicated that the upregulation of GST expression was associated with insecticide resistance. Chen and Zhang [74] found that GSTs play a potential detoxification role in the metabolism of chlorantraniliprole in *Plutella xylostella*. Also, the increase in GST activity in *P. xylostella* enhances its resistance to organophosphates [75,76], Spinosad [77], and pyrethroids [78]. Moreover, GST can detoxify emamectin benzoate in *Oncorhynchus mykiss* [79], *Monocorophium insidiosum* [80], and *Tuta absoluta* [81] and could be a cause of resistance. Furthermore, GST activity is significantly increased against cypermethrin in *Carcinus maenas* [82] and *Spodoptera litura* [83]. Conversely, Fouad, et al. [84] found no correlation between insecticide resistance and CYP-450 activity in two *S. littoralis* strains from Menofya and Fayoum. Likewise, there was no association between resistance and the CarE activities in *H. armigera* [85]. An, et al. [86] reported that rising temperatures are anticipated to suppress the AChE and CarE activities, thereby decreasing the pesticide resistance of *P. xylostella*. Similarly, no clear correlation was found between AChE activity and resistance to beta-cypermethrin, chlorpyrifos, abamectin, and spinosad in *P. xylostella* [87]. In contrast, El-Sayed, Ibrahim, Elsobki, and Aioub [28] showed that the overexpression of CYP-450 and CarE is related to *S. littoralis* resistance to cypermethrin and Spinosad. In addition, enhanced activities of CarE and CYP-450 were linked to indoxacarb resistance in *S. litura* [88]. In summary, understanding the biochemical mechanisms underlying *S. frugiperda* resistance to insecticides is crucial for developing effective strategies to manage and mitigate resistance issues.

## 5. Conclusions

The growing resistance of insects to various insecticides poses significant challenges in agricultural pest control. The susceptibility profile determined in our study will serve as a valuable baseline for monitoring potential changes in susceptibility to the tested insecticides among field populations of *S. frugiperda*. Additionally, further investigations into resistance patterns across different regions in Egypt and other countries must be conducted. Comprehending the biochemical basis of insecticide resistance can aid in developing efficient strategies for resistance management, thus enabling informed decisions regarding the judicious use of insecticides in agricultural fields. In addition, identifying an effective synergistic compound capable of inhibiting detoxification enzymes and increasing pesticide toxicity to insect pests is crucial. The findings of this study demonstrate that the combined use of TPP, DEM, and PBO with cypermethrin, spinosad, and lufenuron showed synergistic effects against *S. frugiperda* larvae in the Fayoum region. Consequently, these findings provide insights into insecticide resistance and the formulation of adaptive strategies for the sustainable management of *S. frugiperda* in Egypt.

## Figures and Tables

**Table 1:** Tested insecticides and their modes of action.

Chemical Group	Common Name	Trade Name	(a.i%) Formulation	Mode of Action *
Organophosphates	Profenofos	Deleron	72% EC	1B
Pyrethroids	Cypermethrin	Sparkel	25% EC	3A
Avermectins	Emamectin benzoate	Proclaim	5%SC	6
Spinosyns	Spinosad	Tracer	24%SC	5
*Bacillus thuringiensis*	Bt	Protecto	9.4%WP	11A
Benzoylureas	Lufenuron	Ferary	10%EC	15
	Diflubenzuron	Difluox	48%EC	15
Diacylhydrazines	Methoxyfenozide	Raner	24%SC	18

* All numbers in the mode of action according to IRAC, 2022.

**Table 2:** Toxicity of two conventional insecticides and three bio-insecticides to a susceptible strain and two field populations of *S. frugiperda*.

Insecticide	Strain	LC_50_ (mg/L) (95% Confidence Limit)	LC_90_ (mg/L) (95% Confidence Limit)	Slope ± SE	χ^2^	*p*-Value	RR
Profenofos	Susceptible	25.66 (19.65–36.24)	100.99 (61.60–277.83)	2.15 ± 0.40	0.07	0.96	----
	Fayum	25.74 (19.28–32.76)	86.002 (61.42–158.09)	2.44 ± 0.42	0.64	0.72	1.003
	Giza	25.76 (18.43–41.84)	154.07 (76.14–896.87)	1.65 ± 0.38	0.29	0.86	1.003
Cypermethrin	Susceptible	5.50 (3.52–7.23)	17.36 (14.31–22.36)	1.53 ± 0.37	0.03	0.98	----
	Fayum	31.70 (22.80–43.56)	152.50 (90.57–499.22)	1.87 ± 0.40	1.14	0.56	5.75
	Giza	20.11 (12.59–46.77)	335.88 (102.82–9259.32)	1.04 ± 0.26	0.66	0.88	3.65
Emamectin benzoate	Susceptible	0.005 (0.0001–0.01)	0.36 (0.08–17.02)	0.63 ± 0.17	0.002	0.95	----
	Fayum	0.003 (0.001–0.007)	0.2888 (0.07–3.63)	0.64 ± 0.12	0.16	0.92	0.58
	Giza	0.001 (0.0004–0.003)	0.05 (0.01–3.53)	0.77 0.20	0.03	0.85	0.20
Spinosad	Susceptible	0.01 (0.002–0.02)	0.75 (0.24–7.45)	0.69 ± 0.14	0.32	0.84	----
	Fayum	0.028 (0.01–0.06)	3.89 (1.10–35.17)	0.59 ± 0.09	053	0.91	2.62
	Giza	0.01 (0.004–0.03)	2.31 (0.43–143.33)	0.58 ± 0.13	1.71	0.42	1.39
*Bacillus thuringiensis*	Susceptible	2519.75 (1766.77–3706.40)	17,953.12 (9465.76–69,208.22)	1.50 ± 0.29	0.46	0.92	----
	Fayum	2895.93 (1853.83–6814.09)	30,569.75 (10,817.78–402,662.86)	1.25 ± 0.28	0.52	0.91	1.14
	Giza	1773.11 (1200.20–2973.36)	13,917.48 (6175.91–141,392.1)	1.43 ± 0.36	0.35	0.83	0.70

Resistance ratio (RR) = LC_50_ of resistant strain/LC_50_ of susceptible strain. Chi-square value (χ2) and degrees of freedom (df) were calculated using Probit analysis (Polo Plus 2.0).

**Table 3:** Toxicity of insect growth regulators (IGRs) to a susceptible laboratory strain and two field populations of *S. frugiperda*.

Insecticide	Strain	LC_50_ (mg/L) (95% Confidence Limit)	LC_90_ (mg/L) (95% Confidence Limit)	Slope ± SE	χ^2^	*p*-Value	RR
Lufenuron	Susceptible	0.08 (0.02–0.22)	33.30 (5.006–3586.91)	0.48 ± 0.11	4.80	0.18	------
	Fayum	0.16 (90.04–0.45)	30.44 (5.14–308.46)	0.56 ± 0.13	1.54	0.46	2.01
	Giza	0.05 (0.02–0.10)	3.23 (1.102–23.19)	0.71 ± 0.12	4.37	0.22	0.65
Diflubenzuron	Susceptible	0.27 (0.16–0.54)	3.44 (1.26–39.57)	1.16 ± 0.26	4.57	0.10	-------
	Fayum	0.35 (0.17–0.72)	10.06 (3.44–77.88)	0.87 ± 0.15	0.95	0.62	1.29
	Giza	0.23 (0.12–0.42)	4.26 (1.66–35.93)	1.02 ± 0.21	0.25	0.87	0.87
Methoxyfenozide	Susceptible	63.01 (47.58–81.84)	231.48 (156.97–482.30)	2.26 ± 0.406	0.53	0.76	-------
	Fayum	64.20 (41.03–126.25)	983.93 (338.08–16914.27)	1.08 ± 0.25	0.40	0.93	1.01
	Giza	69.54 (52.507–88.29)	97.38 (86.12–111.04)	2.82 ± 0.61	0.20	0.65	1.10

Resistance ratio (RR) = LC_50_ of resistant strain/LC_50_ of susceptible strain. Chi-square value (χ2) and degrees of freedom (df) were calculated using Probit analysis (Polo Plus 2.0).

**Table 4:** Synergistic effects of DEM, TPP, and PBO on cypermethrin toxicity to the 2nd instar larvae of Fayoum field population of *S. frugiperda*.

Insecticide	LC_50_ (mg/L) (95% Confidence Limit)	LC_90_ (mg/L) (95% Confidence Limit)	Slope ± SE	χ^2^	*p*-Value	SR
**Susceptible strain**						
Cypermethrin	5.50 (0.001–0.01)	17.36 (14.31–22.36)	1.53 ± 0.37	0.03	0.98	----
Cypermethrin + PBO	4.22 (2.74–5.65)	18.81(12.48–43.76)	1.97 ± 0.404	0.34	0.84	1.30
Cypermethrin + DEM	6.63 (5.03–8.65)	24.55 (16.51–52.21)	2.25 ± 0.41	1.08	0.57	0.82
Cypermethrin + TPP	6.25 (4.61–8.26)	25.51 (16.65–59.49)	2.09 ± 0.39	1.30	0.52	0.88
**Field population**						
Cypermethrin	31.70 (22.80–43.56)	152.50 (90.57–499.22)	1.87 ± 0.40	1.14	0.56	----
Cypermethrin + PBO	18.80 (13.004–29.94)	117.53 (57.31–880.49)	1.61 ± 0.408	0.32	0.84	1.68
Cypermethrin + DEM	22.48 (15.97–37.74)	129.57 (62.86–938.54)	1.68 ± 0.41	0.56	0.75	1.41
Cypermethrin + TPP	21.97 (15.76–3642.61)	128.66 (63.23–756.53)	1.23 ± 0.26	0.05	0.97	1.44

Synergism ratio (SR) = LC_50_ of insecticide alone/LC_50_ of insecticide with the synergist. Chi-square value (χ2) and degrees of freedom (df) were calculated using Probit analysis (Polo Plus 2.0). PBO, piperonyl butoxide; DEM, diethyl maleate; TPP, triphenyl phosphate.

**Table 5:** Synergistic effects of DEM, TPP, and PBO on spinosad toxicity to the 2nd instar larvae of the Fayoum field population of *S. frugiperda*.

Insecticide	LC_50_ (mg/L) (95% Confidence Limit)	LC_90_ (mg/L) (95% Confidence Limit)	Slope ± SE	χ^2^	*p*-Value	SR
**Susceptible strain**						
Spinosad	0.01 (0.002–0.02)	0.75 (0.24–7.45)	0.69 ± 0.14	0.32	0.84	----
Spinosad + PBO	0.015 (0.04–0.003)	6.18 (1.16–213.94)	0.49 ± 0.1003	5.39	0.14	0.66
Spinosad + DEM	0.01 (0.005–0.05)	7.94 (1.43–242.45)	0.48 ± 0.09	3.64	0.30	1.00
Spinosad + TPP	0.004 (0.001–0.011)	0.56 (0.14–9.24)	0.61 ± 0.12	1.40	0.49	2.50
**Field population**						
Spinosad	0.16 (0.07–0.45)	30.44 (5.14–308.46)	0.56 ± 0.13	1.54	0.46	----
Spinosad + PBO	0.028 (0.008–0.09)	8.35 (1.25–675.88)	0.52 ± 0.11	0.34	0.84	5.71
Spinosad + DEM	0.02 (0.005–0.65)	8.24 (1.13–1027.79)	0.49 ± 0.11	0.69	0.70	8.00
Spinosad + TPP	0.02 (0.003–0.1003)	52.03 (2.82–911940.5)	0.37 ± 0.11	0.04	0.97	8.00

Synergism ratio (SR) = LC_50_ of insecticide alone/LC_50_ of insecticide with the synergist. Chi-square value (χ2) and degrees of freedom (df) were calculated using Probit analysis (Polo Plus 2.0). PBO, piperonyl butoxide; DEM, diethyl maleate; TPP, triphenyl phosphate.

**Table 6:** Synergistic effects of DEM, TPP, and PBO on lufenuron toxicity to the 2nd instar larvae of the Fayoum field population of *S. frugiperda*.

Insecticide	LC_50_ (95% CL)	LC_90_ (95% CL)	Slope ± SE	χ^2^	*p*-Value	SR
**Susceptible strain**						
Lufenuron	0.08 (0.02–0.22)	33.30 (21.01–47.41)	0.48 ± 0.11	4.80	0.18	----
Lufenuron + PBO	0.065 (0.009–0.19)	26.36 (3.81–8911.44)	0.49 ± 0.13	0.72	0.69	1.23
Lufenuron + DEM	0.09 (0.02–0.21)	10.27 (2.48–267.84)	0.62 ± 0.13	1.507	0.47	0.88
Lufenuron + TPP	0.148 (0.04–0.37)	15.54 (3.52–466.21)	0.63 ± 0.13	1.006	0.60	0.54
**Filed population**						
Lufenuron	0.16 (90.04–0.45)	30.44 (18.83–53.51)	0.56 ± 0.13	1.54	0.46	----
Lufenuron + PBO	0.14 (0.03–0.04)	44.18 (25.98–100.64)	0.515 ± 0.13	1.06	0.58	1.14
Lufenuron + DEM	0.14 (0.04–0.36)	14.19 (3.08–505.84)	0.64 ± 0.14	0.475	0.78	1.14
Lufenuron + TPP	0.165 (0.05–0.43)	23.41 (4.52–1315.03)	0.59 ± 0.13	1.46	0.48	0.96

Synergism ratio (SR) = LC_50_ of insecticide alone/LC_50_ of insecticide with the synergist. Chi-square value (χ2) and degrees of freedom (df) were calculated using Probit analysis (Polo Plus 2.0). PBO, piperonyl butoxide; DEM, diethyl maleate; TPP, triphenyl phosphate.

## Data Availability

All data and materials are included in the manuscript.
